# Echocardiography in Anderson-Fabry Disease

**DOI:** 10.31083/j.rcm2306201

**Published:** 2022-05-31

**Authors:** Rosa Lillo, Maurizio Pieroni, Antonia Camporeale, Michele Ciabatti, Antonella Lombardo, Massimo Massetti, Francesca Graziani

**Affiliations:** ^1^Department of Emergency Medicine, Fondazione Policlinico Universitario A. Gemelli IRCCS, 00168 Rome, Italy; ^2^Università Cattolica del Sacro Cuore, 00168 Rome, Italy; ^3^Cardiovascular Department, San Donato Hospital, 52100 Arezzo, Italy; ^4^Multimodality Cardiac Imaging Unit, IRCCS Policlinico San Donato, San Donato Milanese, 20097 Milan, Italy; ^5^Department of Cardiovascular Medicine, Fondazione Policlinico Universitario A. Gemelli IRCCS, 00168 Rome, Italy

**Keywords:** Fabry disease, lysosomal storage disorders, cardiac imaging, echocardiography, cardiomyopathy, tissue Doppler, speckle tracking

## Abstract

Echocardiography is the most common diagnostic tool to screen for Fabry cardiomyopathy as it is fast, non-invasive, low-cost, widely available, easily applicable and reproducible. Echocardiography is the first-line investigation, being useful in all the stages of the disease: (1) in gene-positive patients, to unveil signs of early cardiac involvement and allowing timely treatment; (2) in patients with overt cardiomyopathy to estimate the severity of cardiac involvement, the possible related complications, and the effect of treatment. Recently, advanced echocardiographic techniques, such as speckle tracking analysis, are offering new insights in the assessment of Fabry disease patients and in the differential diagnosis of cardiomyopathies with hypertrophic phenotype. The aim of this review is to provide a comprehensive overview on the cardiac structural and functional abnormalities described in Fabry disease by means of echocardiography.

## 1. Introduction

Anderson-Fabry disease (FD) is an X-linked inherited disorder of glycosphingolipid metabolism caused by deficiency of the α-galactosidase A lysosomal enzyme [1]. FD is a rare disease (OMIM 301500) with a reported incidence of 1 in 40,000 to 1 in 117,000 [2], but these data are likely underestimated as screening in newborns showed an incidence of up to 1 in 8882 [3]. Cardiac involvement is common and represents the main cause of impaired quality of life and death in FD patients [4, 5]. Fabry cardiomyopathy (FC) is a pan-cardiac disease as globotriaosylceramide (Gb3) accumulates in the lysosomes of all cardiac cellular types, including cardiomyocytes, conduction system cells, and valvular fibroblasts [6, 7]. The main expression of FC is left ventricle hypertrophy (LVH) that can be indistinguishable from classic hypertrophic cardiomyopathy (HCM) [2].

Echocardiography is the most common diagnostic tool to screen for FC as it is fast, non-invasive, low-cost, widely available, easily applicable and reproducible. All the above make echocardiography the first-line investigation, allowing to (1) monitor the disease in gene-positive patients, unveiling signs of early cardiac involvement and allowing timely treatment; (2) estimate the severity of cardiac involvement in patients with overt cardiomyopathy (3) assess disease progression and possible complications (worsening of systolic or diastolic function, development of obstructive form); (4) monitor the effect of treatment. Moreover, even if no FC pathognomonic echocardiographic sign exists, a comprehensive echocardiographic evaluation, along with clinical and electrocardiographic data following the “red flags” approach [8], can rise the suspicion of FC, helping in the differential diagnosis among cardiomyopathies with hypertrophic phenotype [9].

**Fig. 1.**
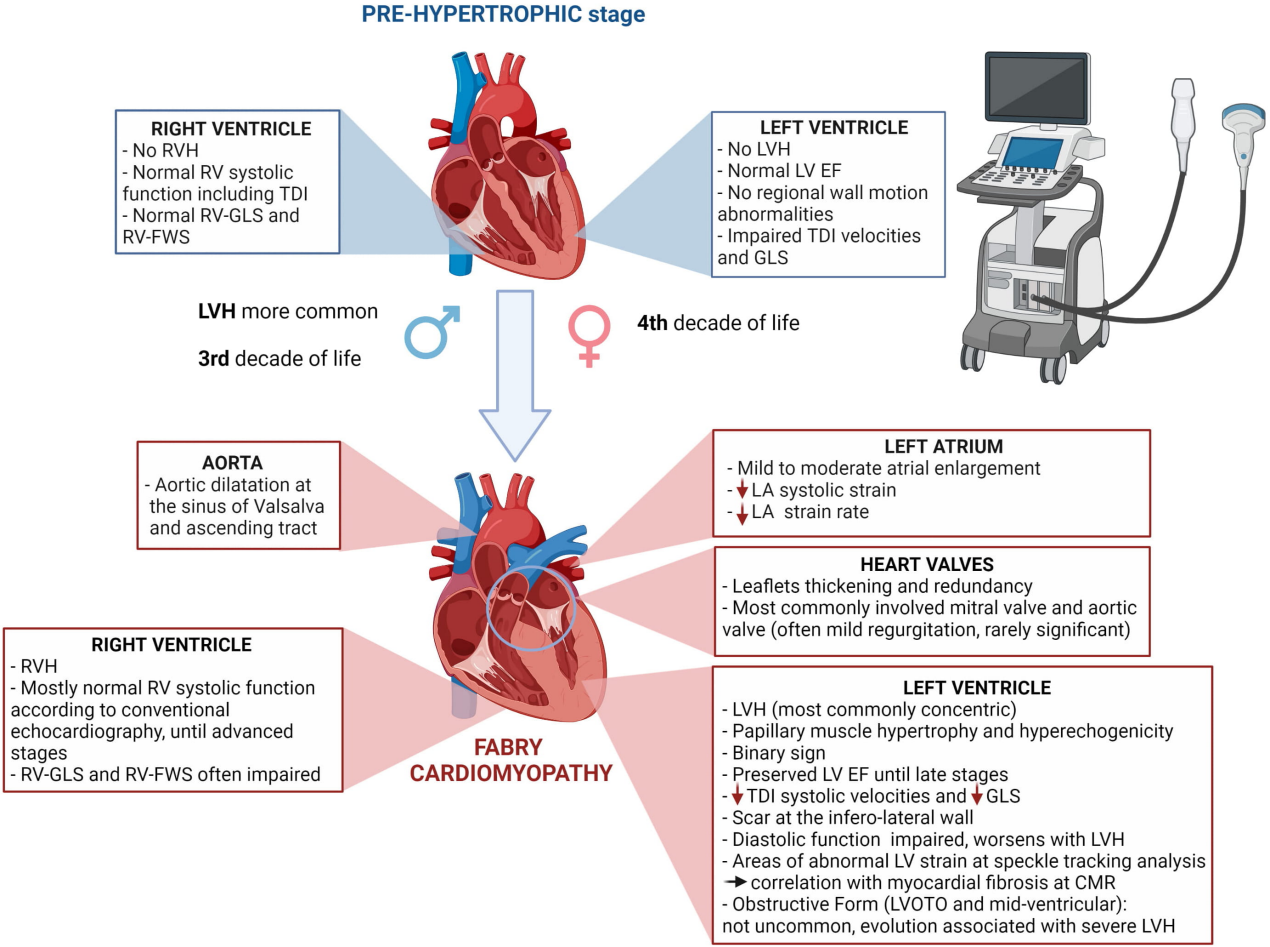
**Main cardiac abnormalities described in Fabry disease by means of echocardiography in pre-hypertrophic stage and overt cardiomyopathy**.

This review provides a comprehensive and updated overview on the cardiac structural and functional abnormalities described in Fabry disease by means of echocardiography (Fig. 1). We summarized what the cardiologist should know, including not only the typical echocardiographic findings of the overt cardiomyopathy but also those that could be clues to unveil the early signs of cardiac involvement.

## 2. Left Ventricle

### 2.1 Left Ventricular Hypertrophy

FD represents an under-recognized cause of LVH and is often misdiagnosed, especially the “cardiac variant” which lacks the typical extra-cardiac findings [10].

Several studies showed that in patients initially diagnosed with HCM the prevalence of FD can vary from 0.5% up to 12% [11, 12]. Therefore, the algorithm of differential diagnosis for patients with unexplained LVH should always include FC, especially when they present as late-onset disease. The best strategy to augment detection of FD is based on an integrated clinic and multi-modality imaging approach. The clinical assessment should not be restricted to cardiological examinations since some cardiomyopathies are manifestations of systemic disorders and those that could be considered “comorbidities” are signs that must rise the suspicion of a specific disease. The recognition of clinical and instrumental ‘red flags’ [8] in the setting of unexplained LVH must guide rational selection of further diagnostic tests including genetic analysis for FD [13].

According to current guidelines, in genetically proven FD patients, LV wall thickness >12 mm, not explainable by other causes, is a marker of cardiac involvement and a criterion to start enzymatic replacement therapy (ERT) (Class I of recommendation) [14].

**Fig. 2.**
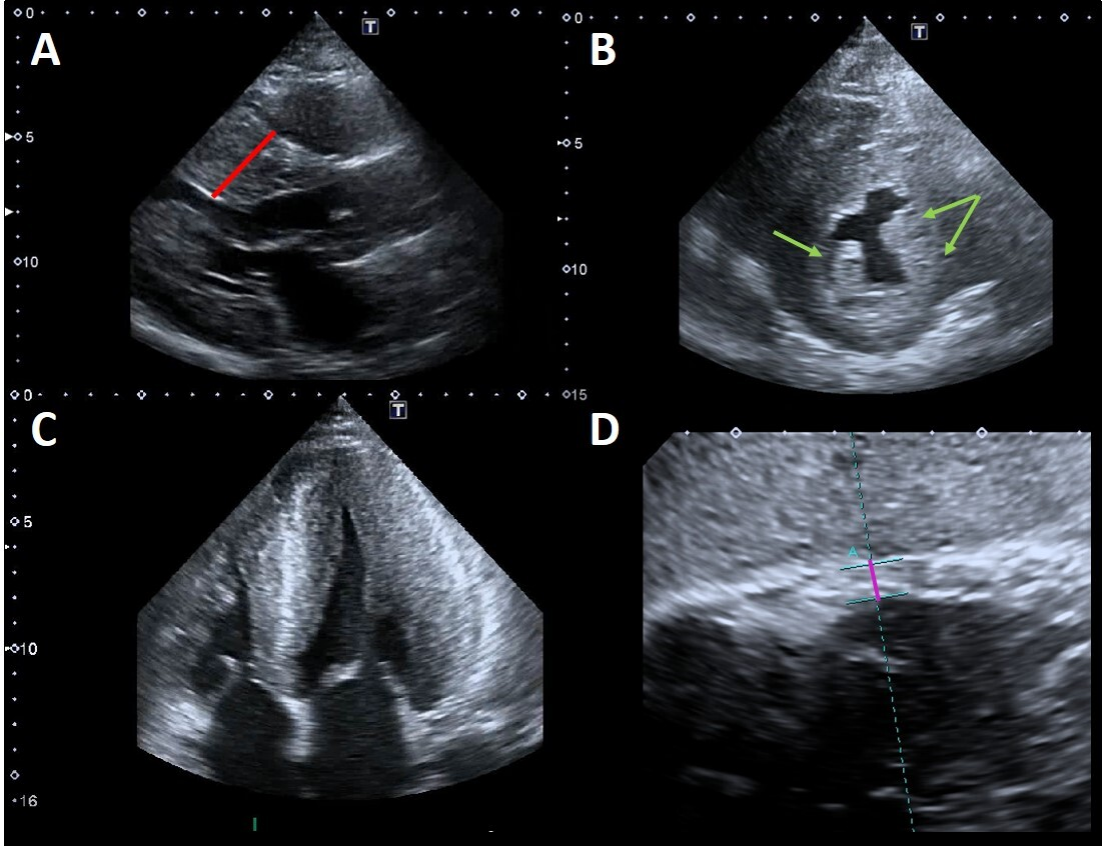
**Example of advanced Fabry cardiomyopathy**. (A) Parasternal long-axis view: the red line shows the maximal wall thickness (30 mm). (B) Parasternal short-axis view: the green arrows point to the prominent hypertrophic papillary muscles. The anterolateral papillary muscle is also bifid (double arrow). (C) Apical four chambers view showing severe concentric LVH with reduced LV cavity dimensions and moderate right ventricular hypertrophy. (D) Subcostal view: the violet line shows the measurement of right ventricular wall thickness (7 mm, n.v. <5 mm).

In FD, LVH is usually concentric [15] (Fig. 2A–C) but FC can also present with eccentric, apical [16], and asymmetric septal hypertrophy [17, 18, 19].

Whatever the distribution of LVH, this results in an increase of the left ventricular mass index (LVMi, defined as >95 g/$m^{2}$ for women and >115 g/$m^{2}$ for men) [20]. LVH is more common in males than females at any given age and this difference was accounted as roughly a decade in a large international FD cohort (714 patients, mean age of patients with LVH 42 ± 14.5 years in men vs 50.1 ± 12.0 years in women) [5].

Sex differences in disease expression are expected, due to the X-linked mode of inheritance of the disease, and clinical FD manifestations in heterozygotes female were considered rare or mild in the past. However, starting from the early 2000s, this paradigm has been challenged by accumulating evidence indicating that females are affected more commonly than previously described [21, 22, 23], with the severity of LVH strongly correlated with increasing age [21].

Wu *et al*. [24] analyzed the cardiovascular manifestations of untreated FD patients with the aim to define the relationship between disease severity, α-GAL activity and cardiac findings. Males with low α-GAL activity and concomitant renal disease were those with most severe LVH, but the latter was present also in females older than 20 years and their log-corrected plasma α-GAL activity levels were inversely correlated with LVMi.

It is worth noting that in patients with other concomitant conditions causing increased LV afterload, it can be difficult to discriminate LVH etiology. In these cases, a comprehensive echocardiographic assessment looking also to functional parameters (i.e., Tissue Doppler and speckle tracking echocardiography) along with other imaging tools such as cardiac magnetic resonance (CMR) are keys for an optimal management. The development of LVH is associated over time with symptoms of heart failure and episodes of arrhythmia (bradyarrhythmia, conduction block, and ventricular tachycardia) [5]. In the largest longitudinal study conducted so far, in which almost 3.000 FD patients were observed before the start of ERT, systemic hypertension and LVH were the strongest predictors of major cardiovascular events, including cardiac-related death [25].

### 2.2 Left Ventricular Morphologic Abnormalities

Some morphologic LV abnormalities have been described in patients with FD, among which the “binary sign” and prominent papillary muscles [9].

The binary sign is the appearance of a clear black and white interface of the LV myocardium due to the adjacency of a bright, hyperechogenic region to a relatively low echo intensity region. This was firstly described by Pieroni *et al*. [26] in a study aimed to identify non-invasive imaging hallmarks of FD comparing echocardiographic features of patients with FC, HCM, and LVH secondary to arterial hypertension. They found that binary sign was present in as much as 83% of FC patients. The match of echocardiography with histologic and ultrastructural findings demonstrated that the binary appearance reflects an endomyocardial glycosphingolipids compartmentalization. Indeed, the hyperechogenic component consists of a thickened glycolipid-rich endocardium, followed by a subendocardial empty space containing free glycosphingolipids and an inner severely affected myocardial layer, with a subendocardial-midwall layer gradient of disease severity in which the hypoechogenic component represents myocardial layers that are relatively spared from glycolipid storage. This finding triggered further research, which contrariwise demonstrated a much lower prevalence of the binary sign (roughly 20%), questioning its value as a screening marker of FD [27]. However, the higher occurrence of this sign in patients with overt cardiac involvement and advanced disease, may partially explain the discrepancies observed among studies enrolling patients with different degree of LVH [27, 28] and nowadays the presence of this sign in Fabry patients in the pre-hypertrophic stage is still unknown. In conclusion, even if the binary sign is not an “infallible diagnostic hallmark”, it may have a value in rising the suspicion of FD in the differential diagnosis of unexplained LVH.

Papillary muscles hypertrophy and hyperechogenicity have been described in FD (Fig. 2A–C) and they might contribute not only to the increased LV mass but also to the development of mitral regurgitation. Niemann *et al*. [29] investigated the diagnostic value of this findings in a study on 101 consecutive patients with concentric LVH of various etiologies (FD, Friedreich ataxia, isolated arterial hypertension, amyloidosis) vs healthy control subjects. Enlarged absolute papillary muscle area was evidenced in 75%, and increased PM_LV_ratio (ratio of papillary muscle size to LV circumference) was found in 78% of 28 FD patients. Nevertheless, also this sign does not allow to certainly discriminate FD from other etiologies of LVH.

Non-compaction and apical aneurisms have also been described in FD, but their meaning is not clear yet [30, 31, 32].

### 2.3 Obstructive Form

Unlike HCM, resting left ventricular outflow tract obstruction (LVOTO) has been rarely described in FD, and it has been long considered an exclusion criterion for FC. However, in the last years a growing number of cases of FC with LVOTO or midventricular obstruction have been described [33, 34, 35, 36, 37, 38, 39, 40]. Calcagnino *et al*. [38] reported a case series of FD patients with drug-refractory exertional symptoms in which exercise echocardiography revealed provocable LVOTO, caused by effort-related reduction in cavity size and papillary muscle hypertrophy. Cecchi *et al*. [39] firstly reported FC diagnosis whose suspicion was raised by the cardiac surgeon during surgical myectomy for obstructive hypertrophic cardiomyopathy, based on the peculiar yellowish appearance of the myocardium with spongy consistency, in patients with no other cardiac and noncardiac FD red flags. These evidences highlight the key role of exercise echocardiography to unveil latent LVOTO when a patient complaints dyspnea as well as the importance of suspecting FD even if the clinical picture suggests HCM.

We recently described the evolution over time of severe FC towards a midventricular obstructive form in 3 patients [40]. In our experience, this evolution occurred in men with the classic form, significant diagnostic delay and severe LVH before ERT initiation. We proposed that this newly described cardiac phenotype could represent an adverse outcome of the disease [40].

### 2.4 Left Ventricular Functional Impairment—Conventional Echocardiography

**Fig. 3.**
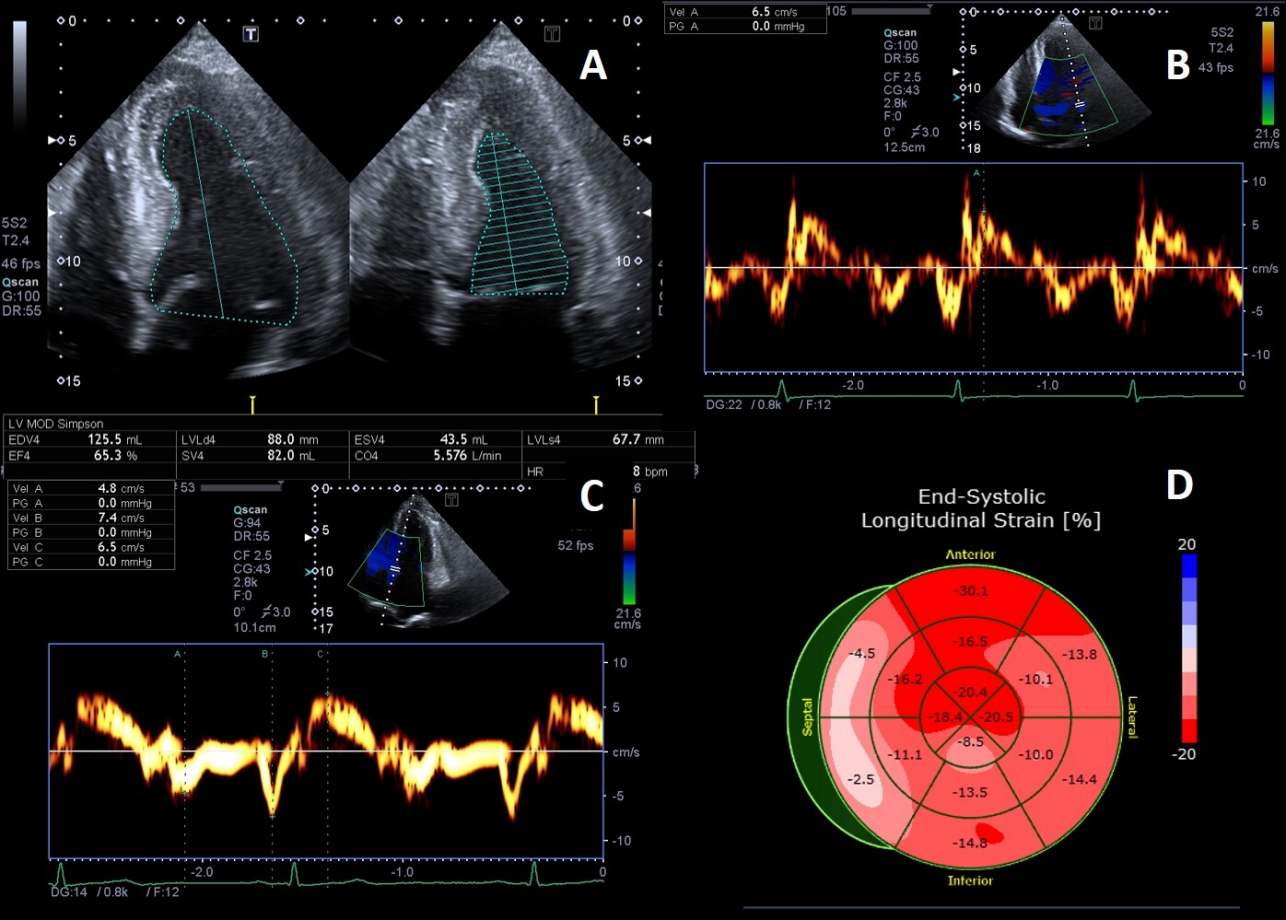
**Example of Fabry cardiomyopathy with normal LV ejection fraction (LVEF) but impaired indexes of longitudinal systolic function**. (A) Apical four chambers view: LVEF 65% measured by biplane Simpson method; (B, C) Tissue Doppler mitral annular velocities at lateral and septal corners respectively, showing low systolic velocities (6.5 cm/s at both sides); (D) 2D speckle tracking analysis bull’s-eye plot, showing reduced LV-GLS value (–15%).

LV systolic function, as measured by ejection fraction (EF), is generally preserved in patients with FD until advanced stages of the disease (Fig. 3A).

However, longitudinal systolic function [41] can be affected in the early stage of cardiac involvement (Fig. 3B–D). Most FD patients without LVH undergoing echocardiography are gene-positive patients in whom the presence of cardiac involvement needs to be addressed. The distinction between “overt Fabry cardiomyopathy” (i.e., LVH) and latent cardiac involvement represents a crucial issue with strong impact on the therapeutic management, as guidelines and recommendations on FD suggest starting ERT as soon as *any* evidence of organ damage is detected.

Pieroni *et al*. [41] compared Tissue Doppler (TD) velocities of patients with Fabry mutations plus LVH (genotype positive/phenotype positive) vs patients with Fabry mutations without LVH (genotype positive/phenotype negative) and healthy controls. They showed that both systolic and diastolic TD velocities were statistically significant lower in patients with Fabry mutation without LVH compared to healthy controls, suggesting that decreased values of both lateral and septal S’ and e’ have a high sensitivity and specificity for identifying Fabry patients without LVH. The specificity and sensibility of these findings were partially confirmed lately [42] but it is now clear that impaired TD velocities are useful to differentiate gene-carriers of hypertrophic phenotypes vs normal or athlete’s heart [43].

Strain rate imaging, a TD-derived technique, is a more sensitive tool to assess myocardial function. Weideman *et al*. [44] found that radial, longitudinal systolic strain and peak systolic strain rate are impaired in FD. In another study, Weidman *et al*. [45] found that FD patients initially show systolic strain rate abnormalities that include decreased longitudinal function, involving the lateral wall first and the septal wall after. The increase in LV wall thickness accompanies the decline in radial function . When myocardial fibrosis is detected at CMR, this is associated with progressive deterioration in longitudinal and radial function. Moreover, myocardial fibrosis correlates with the “double peak” sign, a peculiar pattern that consists of a sharp first peak in early systole, followed by a rapid fall of strain rate near zero, and finally a second peak during isovolumetric relaxation (even if this is not pathognomonic for FD) [46].

FD is characterized by coronary microvascular dysfunction (CMD) that can occur before LVH development [47] and is correlated to the severity of myocardial storage, being worst in patients with LVH [48, 49]. Stress echocardiography is rarely used in the assessment of inducible myocardial ischemia in patients with hypertrophic phenotypes, due to the low sensitivity and the potential harm of dobutamine or other stressors. However, it is worth noting that the echo finding of akinesia and thinning of the inferolateral basal wall [50, 51], often encountered in the late stages of the disease, could be due to repetitive ischemic insult from severe CMD.

### 2.5 Left Ventricular Functional Impairment—Speckle Tracking Echocardiography

Two-dimensional speckle tracking-echocardiography (STE) is a novel echocardiographic technique that has gained increasing popularity in the last years, thanks to its ability in providing objective quantification of cardiac function [52]. STE is an imaging modality based on the assessment of myocardial deformation, through the analysis of “speckles” during the cardiac cycle. STE offers additional advantages over TD, thanks to its ability to assess regional function in all myocardial segments and the reduced angle dependence of measurements [52].

LV strain impairment has been documented in FD patients in all stages of cardiac involvement, with a marked reduction of global longitudinal (GLS) (above all in basal segments) [53] and circumferential strain (CS) [54]. Studies on patients in pre-hypertrophic phase showed that LV GLS is impaired as compared to controls, and specifically an early compromission of longitudinal basal-lateral strain has been documented [55]. Labombarda *et al*. [56] performed a study on LV mechanics investigating base-to-apex longitudinal and CS gradient (defined as the peak gradient difference between averaged basal and apical strain) in FD patients with and without LVH, HCM and healthy controls. They found that LS gradient did not differ between FD without LVH vs controls and between FD with LVH vs HCM patients. Conversely, the CS gradient was lower in pre-hypertrophic FD compared to controls, and lower in Fabry patients with overt cardiomyopathy compared to HCM. They proposed these results as helpful to identify early myocardial involvement in FD and in the differential diagnosis of FC vs HCM. However, the value of these parameters in large real-world cohorts has yet to be determined.

Vijapurapu *et al*. [57] investigated the relationship of early mechanical dysfunction and sphingolipid storage by means of CMR in FD. Intriguingly, they found that in the early stage of the disease (genotype positive-LVH negative), LS impairs as native T1 reduces (i.e., areas of storage), while in FD with LVH, myocardial strain reduces with hypertrophy, storage (detected by a low T1), ECG abnormalities and scar (assessed by late gadolinium enhancement -LGE - ).

Kramer *et al*. [58] confirmed the link between strain and myocardial fibrosis, by finding a correlation between areas of LV strain impairment with those of LGE, suggesting that STE can be used as a tool for the indirect assessment of fibrosis in FC.

### 2.6 Diastolic Function and Left Atrium

Diastolic function is commonly impaired in FC and it worsens with increasing LV wall thickness and fibrosis [9, 59]. However, restrictive pathophysiology is rarely observed, except in the most advanced stages of the disease. A possible explanation of why restrictive physiology is a feature of infiltrative disorders such as amyloidosis but not common in FD may underlie in the different pathophysiology, as amyloid deposition occurs in the interstitium, whereas glycolipid storage occurs intracellularly in FD [9].

In FD patients with or without LVH, the values and the ratio of early diastolic to late diastolic TD velocities are reduced, and the E/e’ ratio is higher compared with healthy controls [41]. Toro *et al*. [42] also demonstrated that isovolumetric contraction time <105 msec had an excellent capability for discriminating patients with pre-clinical stage FD, with a sensitivity of 100% and a specificity of 91%. Furthermore, strain rate during isovolemic relaxation (SRIVR), and the ratio of the peak transmitral E wave to SRIVR (E/SRIVR) demonstrated to effectively differentiate FD patients from healthy controls irrespectively of LVH, with SRIVR <0.235 $\sec^{-1}$ showing sensitivity of 94% and specificity of 92% [60].

Diastolic dysfunction seems closely linked to myocardial fibrosis in FD. Specifically, Liu *et al*. [61] demonstrated that septal ${\mathrm{E/e}}^{\prime }$ ratio is the best echocardiographic parameter associated with prevalence of LGE at CMR. However, as underlined by the authors, diastolic dysfunction is not a prerequisite for LGE in FD, since LGE can precede measurable functional impairments as very early marker of cardiac involvement, above all in females.

Glycolipid storage has been histologically described in atrial myocardium and this correlates with left atrial (LA) dilation, dysfunction and arrhythmias [5]. Atrial enlargement is often at most mild to moderate and the prevalence of LA enlargement is approximately 30%. In cohort studies FD patients have echocardiographic mean LA size greater than age-matched control subjects, but in some cases LA structure and function can remain relatively normal [62]. LA dilatation correlates with LV mass and myocardial fibrosis, and beyond LA myopathy also diastolic function has a role, as demonstrated by increased atrial reversal velocities and duration (i.e., increased LA pressures) [41].

Recent studies on LA speckle tracking analysis offered new insights in FD related LA functional impairment. Boyd *et al*. [63] demonstrated that LA volume is increased in FD patients, even in the absence of LVH. Importantly, in patients without LVH and with LA enlargement, diastolic function can be normal (E’ velocities similar to those of controls) and LA systolic strain and early diastolic strain rate are selectively lower in FD patients with LVH, reflecting reductions in LA and LV relaxation respectively, consequent to increased LV mass. However, regardless of LVH, both FD groups had significant reductions in systolic strain rate and increased LA stiffness index, suggesting that FC may not only cause LVH and fibrosis but can also alter atrial myocardial properties in the early phase of disease process. In line with these findings, Pichette *et al*. [64] showed reduced LA reservoir, conduit, and contractile functions manifested by decreases in peak positive and late diastolic strain by STE. In a comparative study between HCM vs FC patients, the former showed larger LA volume but both disorders had a severe decrease in LA function evaluated by means of STE. Moreover, also in the absence of significant LA dilatation, FD patients can show lower peak atrial LS compared to controls, and an inverse association between peak atrial LS and presence of central nervous system white matter lesions has been documented, suggesting a possible parallel early involvement of heart and brain [65].

All these findings support the hypothesis of an atrial myopathy, which can be independent of LVH and diastolic dysfunction.

## 3. Right Ventricle

Right ventricular (RV) involvement is a common finding in FC (Fig. 2D). Prevalence of right ventricular hypertrophy (RVH) varies between 31% and 71% [66, 67] and its presence correlates with increasing age, disease severity and LVH. Kampmann *et al*. [66] found RVH in 46/129 patients (35.7%) and the 28.2% of them had severely depressed RV systolic function. On the contrary, Palecek *et al*. [68] found a prevalence of RV systolic dysfunction as low as 4.3% among FD patients with RVH. Accordingly, in a study on 45 FD patients, we found that RVH does not seem to significantly affect RV systolic function [69]. Even though RV TD systolic velocity values were slightly lower in patients with than in those without RVH, all parameters of RV systolic function were within the normal range. We also found that RV involvement parallels LV structural changes, being a feature of advanced disease, as supported by the fact that RVH was documented only in patients with concomitant LVH and was associated with LVMi and the Mainz severity score index. Moreover, when compared with patients with amyloid light chain cardiac amyloidosis with similar degree of RVH, patients with FC showed better RV systolic function.

Limited data are available so far for the role of STE in RV analysis. Morris *et al*. [70] found that patients with FD had worst RV systolic function parameters than healthy controls, with RV systolic dysfunction unveiled in 20% of a study population of 50 patients. In this study, a correlation between RV strain abnormalities and RV myocardial fibrosis at CMR was also demonstrated. We recently performed a comprehensive RV STE study, comparing FD patients with vs those without LVH and healthy controls [71]. We found that strain of RV free wall (RV-FWS) and of the free wall + septum (RV-GLS) can be impaired in Fabry patients, even when conventional echo parameters are within normal ranges. Indeed, we found impaired RV-FWS and RV-GLS in as much as 41% and 35% of the overall Fabry population and this percentage rose to 58% and 54% when considering only patients with Fabry cardiomyopathy (according to the normal cut off values of –23% and –20% proposed by Muraru *et al*. [72]). On the other hand, RV strain was preserved in FD in the pre-hypertrophic stage and the physiologic difference between RV-FWS and RV-GLS (Δstrain), a parameter describing the equilibrium of RV mechanical properties, was maintained in FD regardless of the presence of overt cardiomyopathy.

Nowadays few data on the clinical implications of RV involvement in FD are available. We recently investigated the possible association of RVH and RV systolic function with major clinical events in FD [73]. Our data showed that both RVH and RV systolic function were associated with clinical outcome in FD at univariate analysis, but only proteinuria and LVMi emerged as independent predictors of major events. These data corroborate the hypothesis that RVH and RV systolic impairment are markers of advanced disease but do not affect outcome per se.

## 4. Valvular Heart Disease

**Fig. 4.**
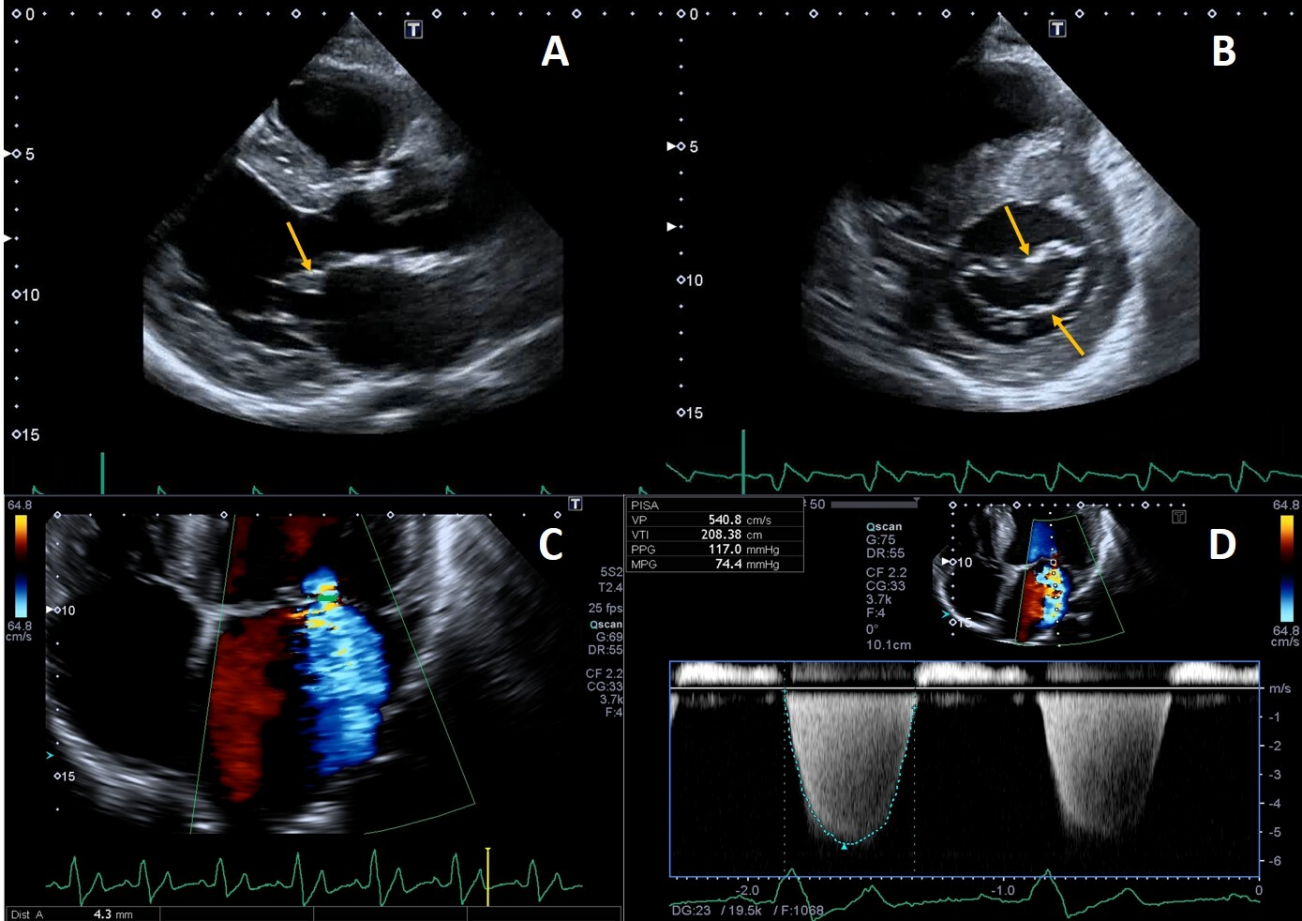
**Example of valvular heart involvement in Fabry Cardiomyopathy**. (A, B) Parasternal long-axis and short-axis views showing thickened mitral valve leaflets (arrows). (C) Color Doppler four chamber view focused on the mitral valve, the green line represents the vena contracta width (4.3 mm) suggestive of moderate mitral regurgitation. (D) CW Doppler across the mitral valve.

Deposition of glycolipids has been histologically reported in all four heart valves [74]. Leaflet thickening and redundancy are commonly encountered (Fig. 4), affecting the mitral and aortic valves in up to 57% and 47% of patients, respectively [15].

Mild mitral and aortic regurgitation are frequently detected on echocardiography, while moderate or greater valvular regurgitation is rare and infrequently lead to clinically significant outcomes. Mitral valve prolapse was described as a frequent finding in the past [75] but this was not confirmed recently [76], likely due to implementation of stricter diagnostic criteria [9]. We recently described a case of isolated chordal rupture, which occurred *without* valve leaflet prolapse, in a patient with Fabry cardiomyopathy. We speculated that in this case chordal rupture could be due to sub-valvular apparatus storage of glycosphingolipids rather than fibro-elastic deficiency [77]. Valvular stenosis is rarely described, even if a case of rapidly progressive aortic stenosis has been reported, and the author proposed that it could have been caused by severe calcification of the aortic valve, as a consequence of valve thickening due to FD or both processes that finally potentiated each other [78].

However, in the largest cross-sectional study conducted so far on 714 patients from 11 countries, only 14.6% had clinically relevant heart valvular disease, and just three patients in the entire cohort had specific indication for valve surgery [5], unlike other storage diseases [79].

## 5. Aortic Dilatation

Aortic dilatation has been reported among FD features [75, 80] and, as demonstrated by biochemical studies of postmortem specimens, it is the expression of degenerative changes in the aortic media caused by excessive accumulation of glycolipids [81]. In FD males, the prevalence of ascending aorta dilation and aneurysms is higher than women [82], in whom this vascular complication occurs 15–20 years later [83]. Moreover, dilation appeared to be independent from cardiovascular risk factors [83] but patients with an aortic root diameter >40 mm exhibited complex cardiac abnormalities including LVH, suggesting a relation between an advanced FD stage and vascular remodeling. In the largest cohort study assessing structural changes in the aorta of FD patients, dilatation of aortic root at the sinuses of Valsalva and ascending aorta developed in as much as 32.7% and 29.6% of males, and 5.6% and 21.1% of females respectively [83].

Thus, patients with FD should be closely monitored for the presence and possible progression of aortic dilation, even if need for surgery and complications such as dissection and rupture have not been formally reported so far [9].

## 6. Effect of Specific Fabry Disease Therapies on Cardiac Structure and Function

According the current European expert consensus document [84], in men over the age of 20 years and women aged over 30, clinical and echocardiographic re-evaluation should be performed on an annual basis. Echocardiography has strong therapeutic implications, as echo signs of cardiac involvement represent a formal indication to start FD specific treatments and allows to monitor the effect of therapies.

To date, there are conflicting results on the effects of ERT on LVH [85, 86, 87, 88, 89, 90, 91, 92, 93, 94, 95, 96, 97, 98]. Data from the Fabry Outcomes Survey database [96] showed that in patients with LVH at baseline, treatment resulted in a sustained reduction in LVMi after 5 years and a significant increase in mid-wall fractional shortening, while in subjects in pre hypertrophic stage these parameters remained stable.

In other studies, even if impressive amelioration of subjective symptoms was achieved, not much improvement in cardiac changes was observed [85]. The different results yielded by different studies are also likely related to differences in baseline populations characteristics. Indeed, it is widely accepted that the maximum benefit of ERT on heart is achieved when the treatment is timely started (before advanced stages of cardiomyopathy). Germain *et al*. [86] found that the mean interventricular septum and LV posterior wall thickness remained stable and normal in all patients but those who initiated treatment at age ≥40 years, who exhibited significant increase in wall thickness.

Sex differences have also been observed. Metanalysis by Rombach *et al*. [98] showed that regardless of LVH at baseline, LVM remains unchanged or increases in males despite ERT, even if at a slower rate compared to untreated male patients. On the other hand, LVM decreases in ERT treated females with LVH at baseline while it remains stable in females without LVH. As far as it concerns Migalastat therapy, in patients with amenable mutations, this drug resulted in reduced LVM compared with both placebo and ERT [99, 100].

ERT consistently showed beneficial effect on TD and STE parameters, however, whether these improvements have a clinical relevance remains to be proved.

In a prospective observational study on 29 FD patients with no cardiomyopathy at baseline, TD abnormalities developed after a median follow-up period of 2.9 years in 16 of 20 untreated patients (80%) vs only 3 of 9 patients (33%) receiving ERT [101]. Similarly, strain analysis revealed an improvement in regional LV strain after agalsidase-β therapy, specifically end-systolic strain and peak systolic strain rate increased significantly in the posterior wall [44]. ERT has been found also to improve LA strain, and increase in peak LA strain (32% to 38%) [64], however it does not significantly reduce LA size. Data of ERT on RV mass reduction are conflicting and no significant change in RV function with ERT has been observed [67, 102].

## 7. Conclusions and Future Perspectives

Echocardiography has the advantage of being non-invasive, fast, reproducible, highly cost-effective and largely available. For these reasons and based on solid evidence accumulated over the decades, echocardiography remains the first-line investigation for all cardiomyopathies, including Fabry disease. However, echocardiography alone is not sufficient for the diagnosis of Fabry cardiomyopathy, and an integrated clinical and multi-modality imaging approach is always recommended. The implementation of speckle tracking echocardiography, which allows the study of cardiac mechanics, offers promising results for differential diagnosis with other form of cardiomyopathies, early diagnosis of cardiac involvement in gene-carriers and their follow-up as well as for non-invasive, indirect assessment of myocardial fibrosis.

**Fig. 5.**
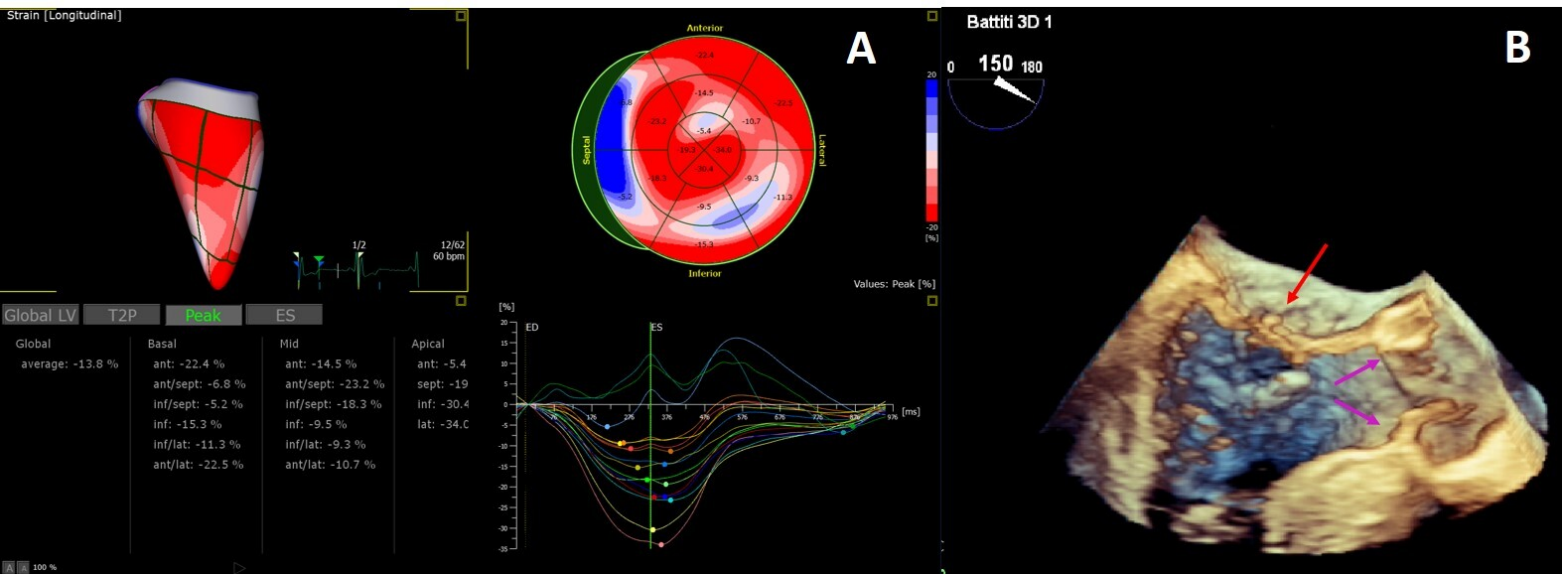
**3D Echocardiography**. (A) 3D echocardiography LV-analysis with regional strain analysis mapped onto the LV model and shown in the bull’s eye plot for clear visualization. (B) 3D transesophageal echocardiogram mid-esophageal long axis view. The arrow points to minor chordal rupture and the purple arrows to mild aortic cusps thickening.

The added value of 3D echocardiography needs to be proved but its strong correlation with CMR for the estimation of LVM and LVEF [103, 104] and for the detailed study of myocardial strain [105, 106] and heart valves [77] (Fig. 5) is promising.

## Abbreviations

FD, Anderson-Fabry disease; FC, Fabry cardiomyopathy; Gb3, globotriaosylceramide; LVH, left ventricular hypertrophy; HCM, hypertrophic cardiomyopathy; LV, left ventricle; ERT, enzymatic replacement therapy; LVMi, left ventricular mass index; LVOTO, left ventricular outflow tract obstruction; EF, ejection fraction; CMR, cardiac magnetic resonance; TD, Tissue Doppler; CMD, coronary microvascular dysfunction; STE, speckle tracking-echocardiography; GLS, global longitudinal strain; CS, circumferential strain; SRIVR, strain rate during isovolemic relaxation; LGE, late gadolinium enhancement; LA, left atrium; RV, right ventricle; RVH, right ventricular hypertrophy; RV-FWS, right ventricular free wall strain; RV-GLS, 6-segment right ventricular global longitudinal strain.
